# The Medical Treatment of Inebriety

**Published:** 1900-05

**Authors:** T. D. Crothers

**Affiliations:** Superintendent Walnut Lodge Hospital, Hartford, Ct.


### THE MEDICAL TREATMENT OF INEBRIETY.

By T. D. CROTHERS, M.D.,

Superintendent Walnut Lodge Hospital, Hartford, Ct.

Inebriety is a more complex disease than insanity. Its progressive degeneration often dates back to ancestors, to defects of growth, retarded development, and early physical and psychical injuries. Later the poison of alcohol, by its anesthetic and paralyzing action, develops more complex states of degeneration, the form and direction of which are very largely dependent on conditions of living and surroundings.

The psychical symptoms show progressive disease of the higher brain centers, both masked and open, with degrees of palsy and lower vitality.

In insanity many definite pathologic conditions are traceable. In inebriety a wider, more complex range of causes appears, the line of march of which is often traceable in more general laws of dissolution. Its medical treatment must be based on some clear idea of what inebriety is, and the conditions present in the case to be treated. This requires a careful clinical study of the symptoms, tracing them back to causes and all the varied conditions formative in the progress of the case. In such a study, heredity appears as the most frequent early predisposing cause.

The question then is, What conditions of life have been most active in developing these inherited tendencies? How can these conditions be checked and prevented? What means and methods are possible in the rational treatment?

The class of cases most commonly noted consists of those due to physical causes. These are the physical and mental strains and drains; also injuries both physical and psychical. The remedies here are distinct, and the means to build up and restore these defects call for therapeutic skill and judgment.

Another class of inebriates seems to be due especially to psychical causes, of which mental contagion of individuals, of conditions and surroundings are most prominent. Here another class of remedies and therapeutic measures are required. These classes are often combined, and the various causes are blended, requiring more accurate study to determine the leading factors in each case. These are conditions which provoke the early use of alcohol, and give form and direction to the progress of the case.

The second part of the clinical study of inebriety is the effect of alcohol. What injury has it caused? How far has it intensified all previous degenerations, and formed new pathological conditions and sources of dissolution? Also what organs have apparently suffered most seriously from the drink impulse? and, most important of all, how far is the use of alcohol a symptom or an active cause?

Having ascertained these facts the medical treatment is the same as in other diseases, the removal of the exciting and predisposing causes, and building up of the body.

The first question is the sudden or rapid removal of alcohol. If the patient is alarmed, and intensely in earnest to abstain, he will consent to have the spirits removed at once. If he is uncertain, and has delusions of the power of alcohol to sustain life, the withdrawal should depend on circumstances. The removal of all spirits at the beginning of the treatment is always followed by the best results. The reaction which follows can usually be neutralized by nitrate of strychnia, $\frac{1}{20}$ of a grain every four hours, combined with some acid preparation. Soda bromide in 50- or 100-grain doses every three or four hours will break up the insomnia and cause sleep the first two nights.

The withdrawal of the spirits should always be followed by a calomel or a saline purge, and a prolonged hot-air or hot-water bath, followed by vigorous massage. Hot milk, hot beef tea, and in some cases hot coffee, are very effectual. If the patient persists in a gradual reduction of the spirits, strychnia, $\frac{1}{20}$ of a grain, should be given every two hours. The purge and hot bath should be given every day while the spirits are used. The form of spirits should be changed from the stronger liquors to wines and beers. Some of the medicated wines are useful at this time, or spirits served up in hot milk. There is no danger of delirium from the withdrawal of spirits, particularly where baths and purging are used freely. The two conditions to be treated at this time are poisoning and starvation. The system is saturated with ptomaines from alcohol, and suffers from defective digestion. The nutrition is impaired, and organic growth retarded. Saline and calomel purges, with baths, meet the first condition, foods and tonics the second. Not infrequently the withdrawal of spirits reveals degrees of brain irritation and exhaustion that are practically manias and delirium, or dementia and melancholia. The essential treatment is to regulate the nutrition and elimination; then arsenic, strychnia, phosphates, iron will comprise the chief remedies that are found most useful.

Many of the chronic cases of inebriety reveal dementia when spirits are removed; others show well-marked paresis or tuberculosis. Symptoms which were attributed to the action of alcohol are found to be due to previous degenerations. In one case the demented talk and conduct while using spirits burst into marked dementia when the drug was withdrawn. In another case the wild, extravagant conduct of the inebriate appears in paresis when free from spirits. The removal of alcohol is often followed by tuberculosis, not suspected before, which apparently starts from some trivial cause and goes on rapidly to a fatal termination. Rheumatism and neuritis are forms of disease which frequently appear after the withdrawal of spirits. Diseases of digestion are common; also diseases of the kidneys. The latter is usually masked, and bursts into great activity when alcohol is removed.

These and many other organic diseases suddenly come into view, and whether they have existed, concealed by the anesthetic action of alcohol, or have started up from the favoring conditions of degeneration caused by spirits, are not known. The therapeutic requirements must reach out to meet all these unsuspected disease states which may appear at any time.

The next question is to ascertain the special exciting causes, and remove or build up against them. In the periodic cases the early favoring causes of the drink storm are often reflex irritations from disordered nutrition, exhaustion, and excessive drains or strains. Later a certain tendency is formed for explosions of deranged nerve energy in alcoholic impulses for relief. This periodicity is often due to causes which can be studied and prevented by remedial measures. In certain cases nutrient and sexual excesses are followed by a drink storm.

In another exposure to malarious influences, where the disease has existed for a long time before, brings on the craze for drink. In other cases constipation, overwork, neglect of hygienic care of the body, irregularities of food and sleep, emotional excitement or depressions are followed by an alcoholic craze. A vast range of psychical causes have been noted. Thus, a residence on the seashore or in high altitudes on mountains provokes this thirst for spirits, and removal to a higher or lower plane is followed by a subsidence of it. Many persons never use spirits except in large cities, or at specially exciting gatherings, or on holidays and festive occasions. Here, evidently, some defect of the brain exists, either organic or functional, which should be reached therapeutically.

Literally, many of the cases have been cured by change of surroundings as well as medicines.

While the ostensible object of medication is to stop the drink craze, this is as far from being curative as the suppression of pain by a dose of opium.

Conditions which cause the disordered nerve force to concentrate in cravings for the anesthesia of spirits are to be neutralized and prevented before a cure can be expected.

The use of narcotics and drugs to check the desire for spirits at the beginning is temporary and is always uncertain. Opium, chloral and cocain given freely at this time often simply change the drink craze for these drugs, which are used in the place of spirits ever afterward.

The return of the drink impulse at regular or irregular intervals is in most cases preceded by premonitory symptoms, which enable the physician to use the preventive remedies. In certain cases calomel and saline cathartics, with prolonged baths, rest or exercise, according to the requirements of the case, have been found curative.

Various cinchona tonics, free from spirits, and iron preparations are often useful. Large doses of strychnine seem more valuable after the full development of the morbid impulse, given when spirits are discontinued. Some of the various coca compounds on the market have had a strong influence in breaking up the drink storm.

In a certain number of cases patients are unconscious of the approach of the drink storm, and are difficult to treat. But when they realize its coming and seek assistance, the task is easier. The general principle of treatment is sharp elimination through all the excretory organs, and the use of mineral tonics, changes of diet and living; particularly a study of the exciting and predisposing causes and their removal. When the drink paroxysm has passed away, then radical constitutional remedies are to be used. The history of syphilis calls for mercury, arsenic and potassium. Defective nutrition requires a study of the diet best suited to build up the tissues.

Entailments from other diseases, as malaria, rheumatism, and various neurotic affections, require appropriate remedies. Tinctures of any form are dangerous. The susceptibility to alcohol is so great that the smallest quantity is felt, although it may not be recognized.

Where spirits are taken continuously the system is always depressed, all functional activity is lowered, and literal palsy and starvation are present.

The removal of alcohol is only a small part of the treatment. The demand for alcohol is a symptom of this progressive degeneration. Giving remedies to produce disgust for the taste of spirits or to break up the cravings for it is not curative. Apomorphia, mixtures of atropia, hydrastin, and a great variety of allied remedies are all dangerous: while apparently breaking up a symptom of the disease present, they often literally increase the degeneration by their irritant narcotic properties and depressing action on the organism. The indiscriminate use of these and allied drugs, in the various specifics for inebriety, is the most dangerous empiricism. It is the same as opium or other narcotics for pain in all cases irrespective of all conditions, and calling the subsidence of the pain a cure. Thus, in some cases a periodic, after a gold-cure treatment, developed into acute dementia, which ended fatally. In others, epilepsy, acute mania, pneumonia, rheumatism, nephritis, followed from the chemical suppression of the drink impulse. In all probability the narcotics used were active, contributing causes to the particular organic diseases which followed.

The masked character of inebriety makes it dangerous to use narcotics beyond a certain narrow limit. Cases which have been subjected to active drug treatment to suppress the desire for spirits are feebler and more debilitated than others. Those who have taken the so-called specifics are marked examples, and, whether they use spirits again or not, are always enfeebled and pronounced neurotics.

In all these cases there is so wide a range of causes and conditions that specific routine treatment is impossible.

Strychnine has recently come into some prominence, and is a useful drug. In some cases where the spirits are withdrawn, its action is pronounced as both a tonic and a stimulant. Given in one-thirtieth grain doses four times a day, for a few weeks at a time, then discontinued, or given in larger doses for a shorter time, the results are usually good.

In some cases certain susceptibilities to the action of strychnia are noticeable, and where the drug is taken to prevent a drink attack, it sometimes rouses it, seemingly precipitating the condition which it is supposed to prevent. This is often anticipated in the muscular tremors and nerve twitchings that evidently come from strychnia when used even in small doses.

Strychnia should never be given alone, except immediately after the withdrawal of spirits. At other times, combined with cinchona or other vegetable tonics, it is an excellent tonic. Care should be used to watch its effects on the motor nerves, and to be sure that the patient is not unusually sensitive to it. Belladonna, atropia, cannabis indica, hyoscyamus, and drugs of this class have a limited value, and should be used with great caution in states of irritation following the withdrawal of spirits. They are best given in combination with other drugs for a brief time and in particular cases. The bromides are valuable in the same way, and in the same conditions, only in much larger doses than mentioned in the text-books. From 50 to 100 grains to a dose are requisite, always accompanied with baths, and never continued more than two or three days. Coal-tar preparations are of uncertain value as narcotics, but may be used in certain cases with good results.

The various mineral and vegetable acids are almost indispensable in selected cases, and often can be given a long time as tonics.

In the treatment of cases, after the paroxysm is over, frequent changes of the form of the tonics are most valuable. Iron, phosphorus, arsenic, potassa, and bitter vegetable tonics should be alternated with free intervals, for periods of months. The various derangements of the system should be watched and treated with appropriate remedies, and every case should be constantly under medical care. The facts of the case having been studied, the question of where the medical treatment can be applied to the best advantage must be determined from the case and its surroundings.

If at home the physician must have full control, and his directions be carried out implicitly. When the drink paroxysm appears, the course of treatment must be prompt and exact. In one case the patient goes to bed, and is secluded from all source of excitement; in another he is sent away to the country and among strangers; in a third case a few days' residence in a hospital or asylum under the care of a physician is sufficient. Hospital treatment, with its exact care and physical and psychical remedies continued for a long time, give the strongest promise of permanent restoration. Wisely adapted medical treatment, based on a careful study of each case, makes it possible for the family physician to treat these cases in the early stages with success.

No single remedy is capable of meeting a wider range of conditions than the Turkish or hot-air baths with free massage. Next to this are hot and cold showers, and hot packs with free rubbing. Bitter tonics and salines with regulated diet are next of importance. Elimination through the bowels, kidneys and skin freely are always essential. Beyond this the good judgment of physicians should determine when to give narcotics and when to abandon them, always remembering their danger and very uncertain temporary action. Also that the cessation of the drink craze is only temporary. If this is accomplished by drug and chemical restraint, the permanency is very doubtful.

The subsidence of the drink symptom by the removal of the exciting causes is the only rational treatment. In this the highest medical judgment possible and the greatest therapeutic skill are essential for success. The medical judgment will determine the exact condition of each case and the possible range of remedies required; not any one drug or combination of drugs; not so-called moral remedies or appeals to the will-power, but a clear, broad, scientific application of every rational means and measure is demanded. A large number of these unfortunate cases are distinctly curable in the early stages, and later, when chronic conditions come on, the possibility of cure continues to a far greater degree than is commonly supposed.

It is the common observation of every one that a certain number of cases recover from the apparent application of the crudest empirical remedies and psychical agencies used in the most unskilful way. This fact furnishes the strongest possible reasons for believing that when inebriety shall be studied and treated as a disease more generally by the profession, a degree of curability will be attained far beyond any present expectation. The present empirical stage of treatment should rouse a greater interest and bring the medical treatment of inebriety into every-day practice. Then the family physician, and not the clergyman and the quack, should be called in to advise.

A new realm of medical practice is at our doors, only awaiting medical study above all theory, and exclusively from the scientific side.

